# Biomechanical Risks Associated with Foot and Ankle Injuries in Ballet Dancers: A Systematic Review

**DOI:** 10.3390/ijerph19084916

**Published:** 2022-04-18

**Authors:** Fengfeng Li, Ntwali Adrien, Yuhuan He

**Affiliations:** 1 Faculty of Sports Science, Ningbo University, Ningbo 315211, China; lifengfeng1999@hotmail.com (F.L.); ntwaliadrien@yahoo.com (N.A.); 2 Savaria Institute of Technology, Eötvös Loránd University, 9700 Szombathely, Hungary; 3 Department of Physical and Health Education, Udon Thani Rajabhat University, Udon Thani 41000, Thailand; 4 CEEC Economic and Trade Cooperation Institute, Ningbo University, Ningbo 315211, China

**Keywords:** ballet dance, foot and ankle injuries, lower limb biomechanics

## Abstract

Professional ballet dancers can be classified as dance artists and sports performers. This systematic review aims to consider the biomechanical risk factors for foot and ankle injuries in ballet dancers, as this could potentially reduce the impact that ‘cost of injury’ may have on ballet companies. An additional outcome was to examine the effects of injury on the career of ballet dancers. This study searched articles in four electronic databases for information in peer-reviewed journals. The included articles examined the relationships between biomechanical factors and the relationship between ballet shoes and foot performance. There were 9 articles included in this review. Among these articles, two focused on the peak force of the foot using two types of pointe shoes, three focused on overuse injuries of the ballet dancer’s foot, one article focused on the loading of the foot of a dancer, and three articles focused on the function and biomechanics of the foot in dancers. This review also found that the pointe shoe condition was the most important factor contributing to a foot injury; overuse injury related to high-intensity training and affected both the ankle and the foot; and metatarsophalangeal joint injury related to the function and structure of the foot. Finally, strengthening the lower extremity muscle is also a recommendation to improve muscle coordination and reduce injuries.

## 1. Introduction

The lower extremities continue to be a research issue in sports sciences [1]. Dancing may be termed a special competitive sport and dancers perform an activity in both open and closed kinetic chains [2]. The open-chain suggests that when performing a movement, the foot is not involved in any weight-bearing activity but moves freely in the air [2]. The closed chain indicates that in a dance movement the foot is subjected to weight-bearing and that all the joints are involved [3]. All dance movements comply with biomechanical rules and laws otherwise there are consequences in terms of injuries, including both chronic and acute injury. Kinetics analysis can also identify injury status using musculoskeletal modeling [4,5,6]. Additionally, in a dance movement, the dancers are required to maintain full ankle plantarflexion and extend through the mid-foot to the toes. This occurs when the foot and ankle are in an abnormal position and increases the flexibility of the ankle [7]. When we consider that foot joints and ligaments are not designed to accept excessive loading, the changes in a dance movement could result in the compression of the soft tissue structure and therefore joint injury [8].

Professional ballet dancers are dance artists, and due to the high-intensity training required and the technical discipline and difficulty needed for dance performance, “dance injury” is common in ballet dancers [9,10,11]. All forms of dance contain highly demanding movements, with an injury incidence of up to 95% over a dancer’s lifetime [12,13,14,15]. Studies have focused on the injury of the knee joint and upper limbs; however, studies are lacking on the intense physical demands of dancing that exposes dancers’ feet to a high risk of injuries such as hallux valgus, metatarsal injury, and subsequent ankle pain [16,17,18,19]. Although clinical examination often points to the underlying cause, injury prevention is often necessary to decrease dance company costs and ensure longevity in the dancers’ careers [20]. As a theatrical performance, ballet is considered a global art [21] requiring dance companies to improve the technique of dancers to provide performances at high technical levels. As a result, injury rehabilitation costs are a significant part of the total expenses for a ballet dance company [22]. The human musculoskeletal system refers to the system having its muscles attached to an internal skeletal system. This structure is crucial for humans to perform complex activities, and because of the complex movements in ballet, injuries are common in ballet dancers. Previous studies have revealed that the injury incidence rate reaches 67–95% in ballet dancers [12,13,14,15,23]. Ballet dancers have a higher incidence of dance injury among all dancers.

**Figure 1:**
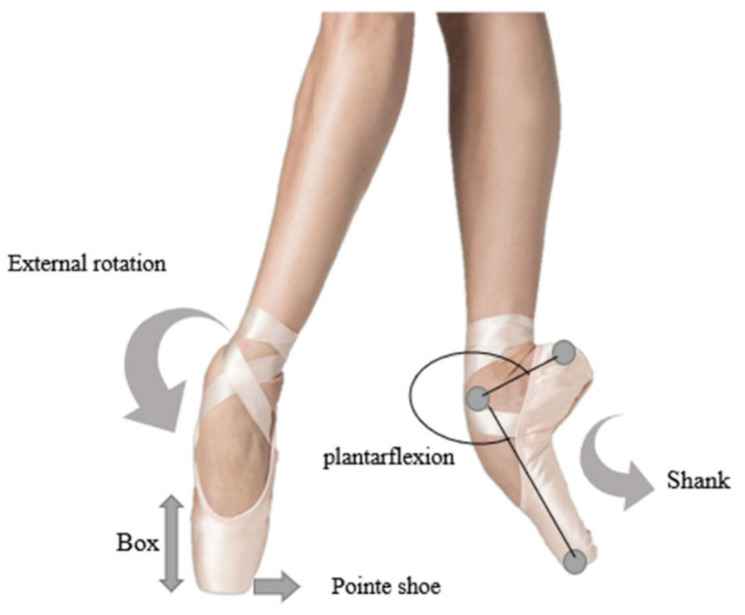
Dancing in the en pointe phase (pointe shoes: the shoes worn by ballet dancers during performance or training; Box: a shoe box that wraps and supports the toes at the front of the shoe; Shank: a piece of rigid shoe bone that reinforces the sole for more support).

Among all the reported injuries, foot and ankle injuries account for a large percentage of all musculoskeletal injuries and are particularly vulnerable to secondary damage suffered by dancers. This is primarily caused by the maximum dorsiflexion or a maximum effort in a turned-out position. For example, the excess force of rotation or turnout of the en pointe (shown in Figure 1) can lead to ankle and foot injuries and cause strain of structures around the ankle [24,25,26]. However, the full extent of the risk factors for foot and ankle injuries in dancers has not yet been summarized. In addition to the upper body and knee injuries, foot- and ankle-related injuries in ballet dancers are high. Foot and ankle function is complicated and needs detailed discussion to determine the mechanisms of injury. It is essential to reach a consensus regarding the benefits of injury prevention among ballet dancers, as this could potentially reduce the impact ‘cost of injury’ may have on ballet companies. Therefore, the purpose of this systematic review was to provide an up to date biomechanical assessment of studies on injury prevention among ballet dancers.

## 2. Materials and Methods

### 2.1. Search Strategy

**Figure 2:**
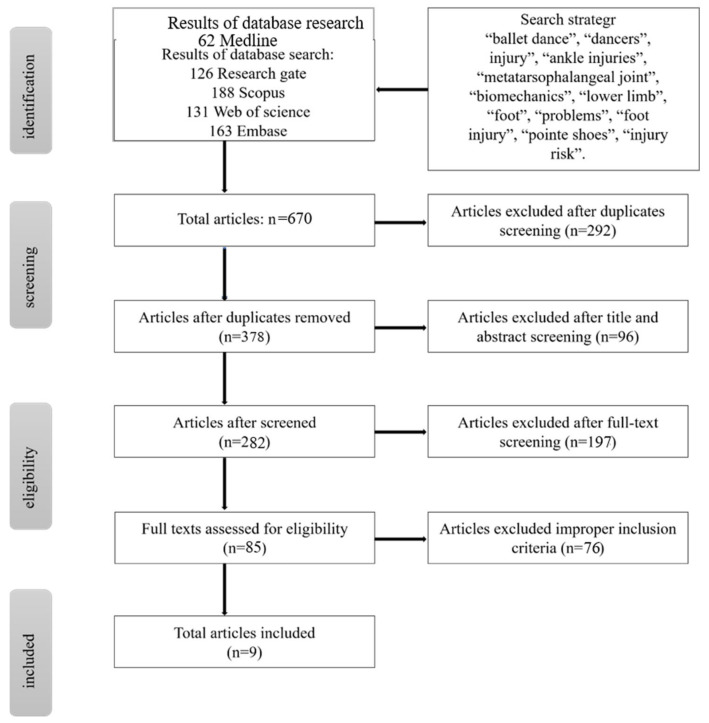
The detailed search strategy of the review.

The authors reviewed the literature published from 1900 through December 2021, on foot and ankle injuries in ballet dance. To identify relevant articles, the research was conducted using five databases: Medline, Web of Science, Web of Science, Research Gate, Embase, and Scopus. The detailed electronic search used the following keywords: “ballet dance”, “dancers”, injury”, “ankle injuries”, “biomechanics injury”, “biomechanics”, “lower limb”, “foot”, “problems”, “foot injury”, “pointe shoes”, and “injury risk”. Among these, “ballet dance”, “foot”, and “injury” were connected with [and]; “biomechanics”, “pointe shoes”, “problems”, and “ankle injury” were connected with [or]. All keywords were searched on each database individually and in combination. Figure 2 shows the detailed search strategy, and Table 1 shows the basic information of the included articles.

### 2.2. Study Selection

Duplicate studies were identified from the different databases and removed. Then, the potential for bias was minimized. Following this, the first two authors independently performed article screening, which included the title, abstract, full-text, and data extraction examination. If disagreement occurred, a third reviewer was identified to review the opinion to reconcile differences.

### 2.3. Data Extraction and Eligibility Criteria

The data were retrieved from the selected articles: the author, publication years, participants’ characteristics, ballet footwear condition, measured variables, purpose, and result. In data extraction, in case of disagreement, another reviewer was included in the discussion to reach a consensus.

### 2.4. Data Eligibility Criteria

The eligibility of selecting papers was estimated according to the following inclusion criteria: (1) the article had to focus primarily on healthy dancer participants wearing pointe shoes; (2) the literature was published in English; (3) the articles focused on the risk factors for the biomechanics of foot injuries in ballet dancers; (4) full articles were based on foot biomechanics injury risk of ballet dancers; and (5) the articles had to be retrievable. When the abstract did not sufficiently present the details for any of the eligibility criteria, the author would browse the full text. The article would be disregarded if it failed to meet the eligibility criteria. The included articles’ characteristics and results are presented in Table 2.

## 3. Results

The bibliographical database search identified 670 citations: 62 in Medline, 126 in Research Gate, 131 in Web of Science, 163 in Embase, and 188 in Scopus. This left a total of 378 articles for evaluation after duplications were removed. Two hundred and eighty-two studies were eliminated following the scanning of the titles and abstracts of the papers. The content of these papers did not reach the required standards for this review. The remaining 85 full-text studies were extracted for detailed review. Seventy-six studies were excluded, as these papers failed to meet the inclusion criteria. A total of nine cross-sectional studies were eventually used and all fully met the inclusion criteria.

Recommendations for decreasing the negative impact of the injury on ballet dancers include three important parameters: (1) shoe condition, (2) overuse injury, and (3) foot biomechanics and function. The investigation on foot and ankle injuries involved biomechanical changes in the lower extremities. Kinematics, such as plantar pressure and peak force in different regions of the foot were investigated. One included study involved the testing of the gait of ballet dancers to compare the difference in foot loading between professional dancers and non-dancers to further analyze risk factors for ballet dancers.

Two studies included several experiments to determine a foot injury for ballet dancers. Based on three different ballet movements (relevé, sous-sus, and pirouette), Aquino et al. assessed the effect of ‘dead’ and ‘new’ pointe shoes on peak forces generated by the foot in professional ballet dancers [27]. The results indicated that the sway area of the swing was significantly higher in the ‘dead’ shoes compared to the ‘new’ shoes. The peak force was significantly higher in the forefoot and hindfoot in the “new” shoe condition when compared to the “dead” shoe condition. Having a higher sway area suggests that a lack of support may contribute to less stability in the foot and ankle joint for ballet dancers when performing routines on their toes. However, there was no difference in peak force between the “new” and “dead” shoes in Bickle et al.’s study. Bickle et al. assessed the differences in the foot and ankle of ballet dancers’ kinetics and kinematics between new and worn pointe shoes [28]. The mid-foot flexion in the “new” and “worn” shoe conditions were significantly different (New: 89.7 ± 5.5°; Old: 96.1 ± 3.9°, p = 0.002, η^2^ = 0.516). The mean results indicated that a significantly greater mid-foot flexion and plantarflexion existed in the worn shoes compared to the new shoes. The mean results indicated that a significantly greater mid-foot flexion and plantarflexion existed in the worn shoes when compared to new shoes. The study also observed that having a greater mid-foot flexion and plantarflexion may be a factor contributing to a higher incidence of ankle and foot injury in ballet dancers [29].

Three studies demonstrated that a fatigue-induced overuse injury is common in ballet dancers. A dancer who requires a long training period has a greater risk of a dance-related injury. In their study, Liederbach et al. concluded that a longer training duration may lead to fatigue, moreover, female athletes were more prone to ACL injury after fatigue [30]. Additionally, Rippetoe et al. determined that a repeated balance technique in the performance of relevé could place atypical stresses and pressure on the joints as well as the tissues of the foot and ankle [31]. The force affected the foot and ankle while performing a dance movement, possibly contributing to injuries among dancers. Previous studies have suggested that around 30% of dancers suffering from acute ankle sprain would develop chronic ankle instability with recurring sprain [32]. Classical ballet requires the dancer to move the ankle between maximum weight-bearing dorsiflexion and maximum weight-bearing plantarflexion. The movements are repeatedly executed during the performance, in daily training, and during rehearsals. Lin et al. mentioned in their research that over-training has been seen as the most common risk factor for fatigue, which would result in impaired movement control and may therefore increase the risk of dance injury [33]. The results suggested that the length of training time in ballet was associated with an increase in the angular values of the left and the right foot, and had a tendency for worsening with increased training time.

Two articles mentioned that understanding the loading distribution strategies of a ballet dancer’s foot is crucial in determining the incidence of foot injury or deformity in the dancer. Prochazkova et al. used a 2 m pressure plate (RSscan International, Olen, Belgium) to collect the gait data of participants (professional dancers and non-dancers) [34]. The results indicated that, compared with non-dancers, the values of the pressure impulse of the big toe and the first metatarsus areas in professional ballet dancers were observed to be significantly higher. The study also revealed that the loading duration of the foot was mainly concentrated on the big toe in ballet dancers. McPherson et al. compared two types of jumping styles (assemble, grand jeté) and three different foot and shoe conditions (flat shoes, pointe shoes, and barefoot) to investigate the maximum ground reaction forces in ballet dancers [35]. The results found that the grand jeté condition had a significantly higher GRFmax than the assemble condition. However, the three shoe and foot conditions showed no significant differences.

Rigidus of the hallux is an arthritic condition of the metatarsophalangeal joint, and excessive pronation of the foot is an important risk factor in the development of hallux valgus. Ballet dancers need 80 to 100 dorsiflexion while performing the act of relevés onto demi-pointe; this is a significantly disabling condition for a ballet dancer. In addition, the stiffness of the foot joint encourages a dancer to roll onto the lateral metatarsals which supports the most bodyweight. Jarvis et al. observed participants performing three different dance movements (relevés, sautés, and saut de chat leaps), and collected data; the article suggested that the focus of relevés is controlling the maximum ankle plantar flexion and metatarsophalangeal joint extension [36]. The greatest range of motion of the metatarsophalangeal joint is required in a ballet dance movement such as relevés. While the peak moment of the metatarsophalangeal joint, observed in the previous study, is small compared to other lower extremity joints, it is essential to consider the metatarsophalangeal joint in a dance injury [37]. Additionally, for the metatarsophalangeal joints, there is a higher demand for ballet dancers than in many other types of movements, which could potentially lead to pain in the foot or damaged toe conditions. Astone et al. examined the differences in the foot while ballet dancers walked compared with non-dancers, and observed that when the feet move, they move with an extra rotation during all walking cycles, reducing the energy expended [38].

## 4. Discussion

To reach a full understanding of the biomechanical risk factors associated with foot and ankle injuries in ballet dancers, knowledge of foot plantar pressure, footwear, and peak force are crucial. This systematic review identified nine articles which appraised either the effect of pointe shoes or overuse on lower limb injuries in ballet dancers. To determine the incidence of foot injury in ballet dancers, it is important to understand ballet position compensatory strategies. This review found that: (1) the pointe shoe condition is an important factor contributing to a foot injury; (2) overuse injury is related to high-intensity training and affected the ankle and foot; (3) metatarsophalangeal joint injury is related to the function and structure of the foot and results in swelling as the demands on the foot increase; and (4) footwear is also related to overuse injuries in professional ballet dancers.

Two studies met the inclusion criteria, the two articles identified the differences between “new” and “dead” pointe shoes. They used different methods, and a consensus emerged that a worn pointe shoe in a dance movement resulted in a significantly increased swing area, especially in the forefoot and mid-foot. Previous studies have found that injuries in ballet dancers can result from inadequate stabilization of the foot and ankle [39]. Pointe shoes are supportive of the foot, which provide stiffness with the compromise of the midfoot ligaments. Having a significant swing in the midfoot area may decrease stiffness and increase the lack of support in the dancer’s foot, increasing the risk of lower limb injury. Previous studies suggested that during some specific dance movements, a repeated impact placed on the foot and ankle may possibly lead to a unique type of injury in a group of dance performers [25,31,40]. Although the peak ankle plantarflexion range of motion was not significantly different between dancers and non-dancers, it should be considered that the plantarflexion function and range of motion of ballet dancers needs measurably exceeded normative values than non-dancers (0 to 50 degrees) [7,41]. Thus, injury prevention is of great significance.

The human foot is an essential element of the locomotor system. It transports the power and the mass of the whole body while in contact with the ground. Ballet dancers use the foot to reach extreme external rotations and the foot and ankle need to twist in the air. As a result, the intense physical demands of dancing put dancers’ feet at high risk of injuries such as hallux valgus, metatarsal injury, and ankle pain [16,17,18,19]. These injuries in a ballet dancer can result in two categories of injury: acute injury, and chronic injury. The structural differences among dancers may change lower limb kinetics and kinematics. The foot has been considered as a triple-arch structure, and the part of the lateral foot performs a rigid arch that supports our weight in a loaded position. Once the weight of the dancer is directed to the mid-foot, there will be consequences for the other parts of the foot. Additionally, to reach an extreme position in ballet, external rotation, especially at the ankle joint, is of great importance for dancers. The limitation of range of motion in the ankle may cause foot pronation (rolling in), and subsequently cause the foot to lose medial arch support. If the stability provided by the mid-foot begins to fail or decrease, dance performance will compensate to maintain the center of mass. Once the performer cannot compensate to keep the body in a correct position the dancer may fall out of the position, which can be the result of an acute injury. Therefore, the evaluation of dancers’ foot injuries and pain should be included in clinical studies, and superimposed X-rays for assessing ankle and foot contributions to the extreme positions required of female ballet dancers offer insight into how these positions are attained [7].

Both the first metatarsophalangeal joint and the ankle are attached to the flexor hallucis longus [25,40,41], and flexor hallucis longus tendinopathy is common in ballet dancers. The metatarsophalangeal joints endure a repeated large range of motion and high peak joint moments may be a risk factor that contributes to the injury of the foot and ankle joints in dancing. Mattiussi et al. suggested that a greater percentage of injuries were classified as overuse injuries [42]. In addition, Shaw et al. noted in their study that older dancers in advanced groups were more likely to be injured [43]. Additionally, the lateral ligament complex of the ankle is the most frequently injured structure of a dancer’s body. In many sports, ankle sprains do not develop into long-term disabilities, however, many patients do not resolve the problem well, which results in residual symptoms persisting for many years. In the continuous training and performance of dancers, repeatedly jumping and landing as well as the extreme plantarflexion or rotation is required. During these dance movements, the repeated shocks and pressure will impact the injured area, and the commonly reported symptoms in dancers include ankle re-injury and instability [44]. All of the articles reviewed mentioned that wearing a pointe shoe and overuse on their foot may accelerate rates of muscle fatigue and ankle sprains [25,45]. Once the forefoot strength decreases, the leg will externally rotate, and the support of the hip joint’s muscle will be not stable when the heel is raised. As a result, all the joint chains will be affected, and ankle and foot re-injury is known to occur in many dancers.

In a ballet dance movement, executing the correct motion such as a simple heel raise of relevé when suffering frequent injuries to the lower limb is painful. Biomechanical analysis evaluating the function of muscles, bones, and tendons is essential in developing diagnostic tools to help identify the causes of injuries for any type of dancer, particularly ballet dancers. The footwear that is needed for each type of dance must also be determined, especially in ballet. It has been suggested that ballet dance footwear manufacturers should consider biomechanical design features in shoe manufacturing, as opposed to just focusing on the aesthetics of the shoes themselves [39]. In support of this, ground reaction force analysis has been seen as a variable of interest because of its potential correlation with increased injury rates. A reasonable design of dance footwear can reduce impact force and improve the stability of a dancer. An emergency stop is a basic technique for a professional dancer, therefor, the muscular lower extremity seems important in daily classes and needs preventive strategies to reduce the risk of injury. Biernack et al. suggested that lower limb strength is also an important factor leading to injuries in the lower extremities [46]. The long-term and intense extreme demands of the musculoskeletal system are relative to plantar pressure distribution in ballet dancers. Therefore, the dancers and dance trainers should reach a consensus that enhancing the controllability of the ankle and foot can reduce ankle restrains to a certain extent. In particular, the flexor hallucis longus and muscle strength enhancement can decrease tenosynovitis probability [47,48]. Dancers also create abnormal dynamic biomechanical forces when using various dance forms [49]. A thorough determination of these forces may inform the physician about the cause of the injures, especially specific overuse injuries. The knowledge provided here is important in the prevention of foot and ankle injuries in ballet dancers. Dance companies need to provide methodologies that reduce the incidence of injury, while improving the teaching quality of dance instruction. We suggest researching the corresponding dynamic effects for pointe shoes in further research. However, there are also some limitations in the current study. First, we focused on the lower limb biomechanics of ballet dancers. Secondly, this review presented some literature that has no specific data, and quantitative assessments cannot be carried out.

## 5. Conclusions

There is substantial evidence for ballet dance injury. Dance medical science, physical medicine, and sports science provide details about the importance of dance injury prevention in dance training. As an art form, ballet dancing requires tremendous physicality, and injury prevention is very important in this discipline. Foot and ankle problems are the predominant pathologies in dance medicine. Therefore, our study systematically reviewed research focused on the risk factors of ankle and foot injuries in ballet dancers. The elements of pointe shoes, overuse of the lower extremity, and the biomechanics and function of the foot are associated with lower limb injury. Improving the design of the shoes to provide stiffness with the compromise of the mid-foot ligaments to reduce injuries seems to be important. However, there are still some limitations in this review; due to the different methodologies used between all the included articles, the data presented in this review are limited. Further analysis is required, focusing on finite element model analysis and electromyography, combined with kinetic and kinematics analysis of the lower limb in ballet dancers. Moreover, we suggest studying the corresponding dynamic effects for pointe shoes in further research. This will provide a better understanding of ballet, and promote ballet dancing to the public while preventing the injury of ballet dancers.

## Figures and Tables

**Table 1:** Basic information of the included articles.

Number	Author/Date	Title	Journal	Concentration
1	Bickle et al., 2018	The effect of pointe shoe deterioration on foot and ankle kinematics and kinetics in professional ballet dancers	Human movement science	Pointe shoes
2	Aquino et al., 2019	Biomechanical Comparison of “Dead” and “New” Pointe Shoes in Female Professional Ballet Dancers	The sport journal	Pointe shoes
3	Prochazkova et al., 2014	Analysis of foot load during ballet dancer’s gait	Acta of Bioengineering and Biomechanics	Foot loading
4	Alyssa et al., 2019	Ground Reaction Forces in Ballet Differences Resulting from Footwear and Jump Conditions	Journal of Dance Medicine & Science	Foot loading
5	Liederbach et al., 2014	Comparison of landing biomechanics between male and female dancers and athletes, part: influence of fatigue and implications for anterior cruciate ligament injury	The American Journal of Sports Medicine	Overuse
6	Lin et al., 2016	Fatigue-Induced Changes in Movement Pattern and Muscle Activity During Ballet Releve on Demi-Pointe	Journal of Applied Biomechanics	Overuse
7	Rippetoe et al., 2020	Multi-Segment Assessment of Ankle and Foot Kinematics during Relevé Barefoot and En Pointe	Pereforming arts foot & ankle	Overuse
8	Jarvis et al., 2016	Kinematic and kinetic analyses of the toes in dance movements	Journal of sports sciences	Foot
9	Astone et al., 2019	Comparison of gait kinematics and kinetics between qualified dancers and non-dancers	Journal of Physical Education and Sport	Foot

**Table 2:** The characteristics and results of included articles.

First Author, Publication Year	Participants (Age: Year; Height: m; Weight: kg)	Experimental Measurements and Purposes	Results
Bickle, 2018	*n* = 15 age: 26 ± 4 height: 1.63 ± 0.61 weight: 51.7 ± 3.8	A 2D video camera, A force platform (Kistler 9287BA Force Platform, Kistler Instruments Ltd., Hampshire, UK), A pedobarograph (RSScan 0.5 m USB2 Plate, RS Scan Ipswich, UK) Purpose: investigating the differences in the kinetic and kinematic of the foot and ankle between a new and a worn pointe shoe.	There is a significantly greater mid-foot flexion and plantarflexion existing in the worn shoes as compared to new shoes,
Aquino, 2019	*n* = 13 age: 20.9 ± 1.9 height: 1.64 ± 0.35 weight: 52.1 ± 5.6	All data using an AMTI force plate collected (sampling rate of 960 Hz) Purpose: Examine the ground reaction forces and center of pressure differences between a “new” and “dead” shoe.	The way area of oscillation was significantly higher in “dead” shoes and the training time of the pointe shoe was related to an overuse injury in female ballet dancers.
Astone, 2019	*n* = 6 age: medial 22.83 height: medial 1.64 weight: 56	An optoelectronic system with six infrared cameras, two force platforms (BTS P6000) Purpose: comparing the gait kinematics and kinetics between professional dancers and non-dancers.	The dance movement they performed caused some differences in their motor skills. Dancers apply for compensation during their gait cycle that makes their gait as effective as possible.
Prochazkova, 2014	*n* = 13 (professional dancers) age: 24.1 ± 3.8 height: 1.70 ± 0.85 weight: 58.3 ± 11.2 *n* = 13 (non-dancers) age: 26.1 ± 5.3 height: 1.73 ± 0.73 weight: 74.1 ± 12.5	the Footscan gait software (version 7.97) purpose: comparing the peak pressure, total loading, and duration of the loading in the selected areas of the foot between the experimental group and control group.	There are greater peak pressure values in the big toe and higher values in the areas of the medial heel in professional dancers. The heel areas have a significantly longer duration of contact with the floor in both two groups.
Liederbach et al., 2014	*n* = 40 (female 20; male 20)	purpose: analyzing dancers and team atheletes’ resistance and its effect on the biomechanics of single-legged landings.	Dancers took longer to reach fatigue, and female athletes are more prone to ACL injury after fatigue.
Lin et al., 2016	*n* = 20 (female) age: 17.98 ± 1.51 height: 1.60 ± 0.57 weight: 49.8 ± 5.4	A motion analysis system (Motion Analysis Corporation, Santa Rosa, CA, USA) Purpose: This article aims to evaluate the impact of fatigue on the performance of ballet dancers	Over-training has been seen as the most common risk factor for fatigue, which would result in impaired movement control and may therefore increase the risk of dance injury.
Rippetoe, 2020	*n* = 11 age: 21 height: 1.68 weight: 55.11	12-camera Qualisys™ Motion Analysis System and AMTI Force plates Purpose: describe the biomechanical differences between the barefoot and en pointe conditions while balancing in relevé and the differences between the barefoot and en pointe shoes.	There is a greater sagittal movement, and a greater midfoot, forefoot, arch height, and rotation movement when a dancer balances barefoot.
Jarvis, 2016	*n* = 10 age: 27.6 ± 3.2 height: 1.60 ± 0.1 weight: 56.3 ± 6.9	An 11-camera three-dimensional motion analysis system, AMTI force plate Purpose: comparing the motion and moments of the metatarsophalangeal joints during three common dance movements.	The peak joint moments related to the dance movement, and the largest values were found during saut de chat leaps, and the smallest value was found during relevés. Many dance movements place high demands on the foot and ankle joints.
Alyssa, 2019	*n* = 21 age: 19.28 ± 1.00 height:1.67 ± 0.44 weight: 52.74 ± 3.42	A recessed force plate (AMTI Accugait System Model ACG, Watertown, MA, USA) with a 2.5 m runway, a video camera (Sony Electronics Inc., San Diego, CA, USA) Purpose: (1) Investigating the maximal ground reaction force when dancer lands from two jump conditions in pointe shoes, barefoot and flat shoes; (2) exploring the specific pointe shoes characteristics effect on ground reaction force.	There is no significant difference in maximum ground reaction force between the three shoe conditions. A significant difference was found between the two types of jump conditions. The jumping distance was greater in the grand jeté but the jumping height was greater in assemble.
